# Genome Sequences of Four Subcluster L2 *Mycobacterium* Phages, Finemlucis, Miley16, Wilder, and Zakai

**DOI:** 10.1128/genomeA.01233-17

**Published:** 2017-11-16

**Authors:** Christopher D. Herren, Alexandra Peister, Timothy S. Breton, Maggie S. Hill, Marcy S. Anderson, Adeline W. Chang, Sydney B. Klein, Mackenzie M. Thornton, Stacy J. Vars, Kasey E. Wagner, Paige L. Wiebe, Thomas G. Williams, Coraima P. Yanez, Jasanta M. Ackles, Darius Artis, Ryan J. Brazier, Ronald J. Bryant, Kerel O. Callwood, Isaiah H. Carter, Cameron L. DeBose, Christian D. Edwards, Isaiah C. Ezemba, Renshal R. Joaquin, Zakai M. Meghoo-Peddie, Ziha G. Meghoo-Peddie, Ryan W. Moore, Christopher E. Smith, Adam J. Turner, Raymond L. Vorters, Jeffrey J. Wider, Liesel L. Krout, Mia S. Comis, Madison J. Davick, Eli E. Michaud, Bailey E. Shevenell, Sarah E. Stanley, Chelsey I. Frank, Jacob R. Montgomery, Lawrence S. Blumer, Jean A. Doty, Martha Smith-Caldas, Welkin H. Pope, Steven G. Cresawn, Daniel A. Russell, Rebecca A. Garlena, Deborah Jacobs-Sera, Graham F. Hatfull

**Affiliations:** a Division of Biology, Kansas State University, Manhattan, Kansas, USA; b Department of Biology, Morehouse College, Atlanta, Georgia, USA; c Department of Biology, University of Maine at Farmington, Farmington, Maine, USA; d Department of Biological Sciences, University of Pittsburgh, Pittsburgh, Pennsylvania, USA; e Department of Biology, James Madison University, Harrisonburg, Virginia, USA

## Abstract

Four subcluster L2 mycobacteriophages, Finemlucis, Miley16, Wilder, and Zakai, that infect *Mycobacterium smegmatis* mc^2^155 were isolated. The four phages are closely related to each other and code for 12 to 14 tRNAs and 130 to 132 putative protein-coding genes, including tyrosine integrases, *cro*, immunity repressors, and excise genes involved in the establishment of lysogeny.

## GENOME ANNOUNCEMENT

Bacteriophages are important research tools for studying basic biological processes, including host-pathogen interactions, and have potential as therapeutic agents in an age of ever-increasing antibiotic resistance (1–3). The Science Education Alliance–Phage Hunters Advancing Genomics and Evolutionary Science (SEA-PHAGES) program seeks to understand these interactions and to collect genome data for future applications (4). Phages exhibit a high degree of gene diversity, even among phages that infect the same host (5). Here, we report the genome sequences of four mycobacteriophages that were isolated, studied, and annotated by undergraduate students as part of the SEA-PHAGES program.

Mycobacteriophages were isolated by enrichment from soil samples at 37°C using *Mycobacterium smegmatis* mc^2^155 as a host. Mycobacteriophages Finemlucis and Miley16 were found in Manhattan, Kansas, USA, and Farmington, Maine, USA, respectively; Wilder and Zakai were found in Atlanta, Georgia, USA. The phages were purified and amplified, and double-stranded DNA was extracted. Each genome was sequenced at the University of Pittsburgh using Illumina MiSeq technology with 150-bp single-end reads. All genomes were assembled using Newbler and verified in Consed. A single contig was obtained for the Finemlucis (77,031 bp), Miley16 (76,653 bp), Wilder (75,806 bp), and Zakai (76,363 bp) genomes, all with >460-fold coverage. All these genomes contain a 58.9% G+C content and have defined ends, with complementary 10-base 3′ single-stranded DNA extensions (right end, 5′-TCGATCAGCC). Protein-coding genes were predicted in DNAMaster (http://cobamide2.bio.pitt.edu/computer.htm) using Glimmer (6) and GeneMark (7). Finemlucis, Miley16, Wilder, and Zakai have 129, 132, 130, and 131 predicted protein-coding genes, respectively, 35 to 39% of which can be assigned putative functions using Phamerator (8), BLASTp, and HHpred (9). Each genome contains either 12 (Finemlucis and Miley16) or 14 (Wilder and Zakai) tRNAs.

All four genomes belong to subcluster L2 mycobacteriophages and exhibit high nucleotide sequence similarity to each other, with substantial pairwise span length coverages (95 to 96%). They are closely related to other subcluster L2 phages (>90%) but are more distantly related to phages in subclusters L1 (66 to 68%), L3 (62 to 66%), and L4 (69 to 73%). The genes involved in virion structure and assembly functions are located in the left arms and transcribed rightward. These are followed by the lysis cassettes, including putative lysin A, lysin B, and holin genes, although the lysis cassettes in Finemlucis and Zakai are different than those in Miley16 and Wilder, with the latter being similar to those in the majority of other subcluster L2 phages (10). The Finemlucis and Zakai lysis cassettes are similar to those in subcluster L2 phages Archie and Rumpelstiltskin, as well as to those in the subcluster L1 phages (10). Predicted immunity repressors, tyrosine integrases, excises (Xis), and putative *cro* genes are present in all four genomes, with repressor and *cro* genes divergently transcribed as in the canonical lambda system (11). Together, these features are consistent with temperate lifestyles for these phages, and stable lysogens were constructed for Finemlucis that are immune to superinfection and spontaneously release phage particles.

### Accession number(s).

The genome sequences of *Mycobacterium* phages Finemlucis, Miley16, Wilder, and Zakai have been deposited in GenBank under the accession numbers MF185728, MF185730, KX580962, and KX580961, respectively.
